# Preserved seasonal variation in glycemic control in patients with type 2 diabetes mellitus during COVID-19: a 3-year-long retrospective cohort study in older adults in Japan

**DOI:** 10.1186/s12902-024-01602-8

**Published:** 2024-05-17

**Authors:** Shimpei Iwata, Kenji Ashida, Mutsuyuki Demiya, Ayako Nagayama, Nao Hasuzawa, Satoko Yoshinobu, Aya Sonezaki, Junichi Yasuda, Seiichi Motomura, Yoshio Katsuki, Kenzo Sugi, Masatoshi Nomura

**Affiliations:** 1 Division of Endocrinology and Metabolism, Department of Internal Medicine, Kurume University School of Medicine, 67 Asahi-Machi, Kurume, Fukuoka 830-0011 Japan; 2 Sugi Cardiovascular Hospital, 950-1 Taguma, Ohmuta, Fukuoka 837-0916 Japan

**Keywords:** Body mass index, COVID-19, Diabetes mellitus, type 2, Lifestyle, Seasonal variation

## Abstract

**Background:**

The coronavirus disease 2019 (COVID-19) pandemic has changed our lifestyle by imposing restrictions, such as physical distancing. The effect of COVID-19 prevalence on seasonal variations in glycemic control in patients with diabetes mellitus (DM) remains unknown.

**Methods:**

This single-center retrospective cohort study evaluated glycemic control in patients with type 2 DM who visited Sugi Cardiovascular Hospital in December 2021. We evaluated the clinical findings of all patients treated regularly between March 1, 2019, and December 31, 2021, including the periods both before and after the COVID-19 pandemic. All the standard treatments were approved. Furthermore, seasonal changes in hemoglobin A1c (HbA1c) levels were evaluated using stratified analyses based on age.

**Results:**

This study analyzed 86 patients (mean age, 69.6 ± 9.2 years; men, 57). Median HbA1c (National Glycohemoglobin Standardization Program [Union of Clinical Chemistry]) levels in spring (March) were 7.70% (interquartile range (IQR):7.23%–8.30%) [60.6 mmol/mol (IQR:55.4–67.2 mmol/mol)], 7.35% (IQR:6.90%–7.90%) [56.8 mmol/mol (IQR:51.9–62.8 mmol/mol)], and 7.50% (IQR:7.10%–8.00%) [58.5 mmol/mol (IQR:54.1–63.9 mmol/mol)] in 2019, 2020, and 2021, respectively. During these periods, HbA1c levels and body mass index (BMI) revealed significant seasonal variations “high in spring” and “low in autumn.” Median HbA1c levels in spring (March) and autumn (September) were 7.86% [61.2 mmol/mol] and 7.48% [57.4 mmol/mol] in 2019 (*P* < 0.001), 7.50% [57.7 mmol/mol] and 7.17% [54.2 mmol/mol] in 2020 (*P* < 0.001), and 7.61% [58.3 mmol/mol] and 7.19% [53.8 mmol/mol] in 2021 (*P* < 0.001). Seasonal variations in HbA1c levels and BMI were maintained over the past 3 years, including the pandemic period. None of the patients in this study developed COVID-19 during the study period.

**Conclusions:**

Seasonal variations in glycemic control in patients with DM were not influenced by lifestyle modifications associated with COVID-19. Maintenance of physical activity is necessary to prevent the development of sarcopenia. Moreover, seasonal variations in glycemic metabolism should be considered an independent factor for DM management. Additional extensive multifacility investigations are necessary to corroborate our findings.

**Supplementary Information:**

The online version contains supplementary material available at 10.1186/s12902-024-01602-8.

## Introduction

Seasonal variations in glycemic metabolism are observed in patients with diabetes mellitus (DM) [1, 2]. The hemoglobin A1c (HbA1c) level is high in winter and low in summer (i.e., highest in March and lowest in September) in patients with type 2 DM in Japan [3, 4]. Decreased physical activity and increased caloric intake in winter contribute to seasonal variations in HbA1c levels [5] and insulin sensitivity [6]. Lifestyle: Food intake (quantity, quality, and frequency), exercise, and sleep have been investigated for their possible association with glycemic control [7, 8], including seasonal variation in glycemic control [9]. Seasonal events and celebrations have the potential to lead to cyclical variations in HbA1c levels [10]. Furthermore, lifestyle indirectly affects various complications of DM by controlling blood pressure, lipid metabolism, and body weight [11]. Thus, lifestyle modifications may affect glycemic control [12] and the management of patients with DM, either negatively by exacerbating the disease or positively by improving it.

The 2016 Kumamoto earthquake in Japan worsened glycemic control in patients with DM, probably through lifestyle modifications [13]. Coronavirus disease 2019 (COVID-19) spread worldwide in 2019; thus, many people were forced to change their lifestyles, particularly because of the lockdowns observed worldwide. The estimated number of COVID-19 patients in Japan was 390,190 on January 31, 2021; however, no lockdown was imposed [14]. However, people were encouraged to avoid closed spaces, crowded places, and close-contact settings (i.e., the 3Cs), maintain physical distancing [15], and limit movement under the terms of the COVID-19-related state of emergency declared between April 7, 2020, and May 25, 2020; January 8, 2021, and March 21, 2021; April 25, 2021, and June 20, 2021; and July 12, 2021, and September 30, 2021, in Japan. Generally, people in Japan had modified their lifestyles in strict accordance with these recommendations.

A recent analysis based on mathematical modeling in India predicted that lockdown measures would cause a substantial increase in HbA1c levels and DM-related complications [16]. Conversely, other studies have reported that lockdown measures do not exacerbate glycemic control in patients with type 1 DM and young adults with type 2 DM [17, 18]. However, the effect of COVID-19 on seasonal variations in glycemic control in patients with type 2 DM remains unclear.

This single-center retrospective cohort study sought to investigate the changes in glycemic control of patients with type 2 DM due to lifestyle modifications caused by the pandemic. In this study, we analyzed the changes in glycemic control of patients with type 2 DM during the pre-pandemic and pandemic periods for 3 years. Clarifying lifestyle modifications based on seasonal glycemic variations in patients with type 2 DM may improve the management of these patients.

## Materials and methods

**Fig. 1:**
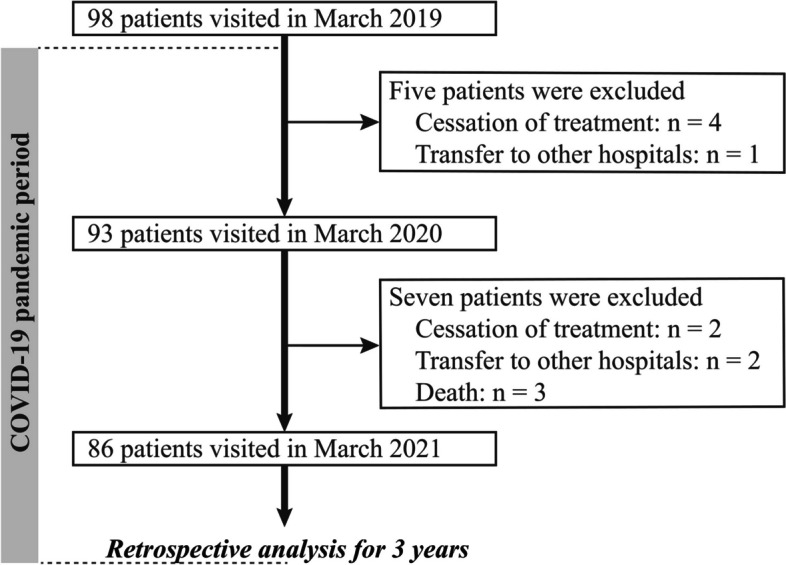
Flow chart of the present study. This study included patients who visited our hospital regularly for three years (between 2019 and 2021)

This study enrolled first a total of 98 patients with type 2 DM who visited the Department of Endocrinology and Metabolism of Sugi Cardiovascular Hospital between March 1, 2019, and December 31, 2021. We excluded five and seven patients from the yearly cohorts of 2019–2020 and 2020–2021, respectively (Fig. 1). Thus, data from 86 patients were analyzed (Fig. 1 and Table 1). Physical and laboratory data were collected every 3 months during the hospital visits between March 1, 2019, and December 31, 2021, for each patient. All procedures complied with the ethical standards of the Institutional Review Board of Sugi Cardiovascular Hospital and the 2013 Declaration of Helsinki. This study was approved by the Ethics Committee of Sugi Cardiovascular Hospital (20,201,207), and the patients provided written informed consent.

**Table 1:** Participant characteristics

Parameters	Values
**Participant number**, N	86
Male, n (%)	57 (66.3)
**Age (years)**, mean ± SD	
Total	69.6 ± 9.2
Men	71.0 ± 9.1
Women	68.0 ± 9.3
**Race**, n (%)	
Japanese	86 (100)
**Duration of DM (year)**, median (IQR; range)	13.5 (9–23; 1–50)
**Current smoker,** n (%)	31 (36.0)
**Alcohol drinker,** n (%)	22 (25.6)
**Low income,** n (%)	0 (0)
**Complications**, n (%)	
Diabetes-related neuropathy	43 (50.0)
Diabetes-related retinopathy	22 (25.6)
Diabetes-related nephropathy	33 (38.4)
Hypertension	57 (66.3)
Dyslipidemia	49 (57.0)
Cardiovascular disease	24 (27.9)
**Lifestyle modifications after COVID-19**, n (%)	
Overall activity	25 (29.1)
Increased food intake	12 (14.0)
Decreased exercise	20 (23.3)

The data of parameters (age, duration of DM, and complications) were obtained in March 2019. Surveillance of patients’ lifestyle modifications after COVID-19 prevalence was performed in December 2021. Data showing normal distribution are presented as means ± SDs or numbers with percentages. The data showing non-normal distribution are presented as median (IQR; range)

Abbreviations: *DM* diabetes mellitus, *SD* standard deviation, *IQR* interquartile range

Physical and laboratory data of the participants, including complications, treatment, body weight, body mass index (BMI), and blood pressure, were evaluated before and after the spread of COVID-19. Hypertension was defined as the requirement for antihypertensive therapy, systolic blood pressure ≥ 140 mm Hg, or diastolic blood pressure ≥ 90 mm Hg. Dyslipidemia was defined as dyslipidemia therapy, low-density lipoprotein cholesterol level of ≥ 140 mg/dL, or serum triglyceride level of ≥ 150 mg/dL. Coronary artery disease was defined as a history of angina or myocardial infarction. The results of laboratory tests, including HbA1c levels, as per the National Glycohemoglobin Standardization Program values (%) with accompanying International Federation of Clinical Chemistry values (mmol/mol) in brackets, and the indicators of DM profile, liver function, renal function, and lipid profile were also evaluated. HbA1c was measured by high-performance liquid chromatography (HPLC) using HLC-723GR01® (Tosoh Bioscience, Yamaguchi, Japan) with a coefficient of variation of less than 2%.

Patients’ diet and exercise changes were surveyed during the COVID-19 period using a written questionnaire in December 2021. Data were compared every 3 months to assess seasonal variation, starting in March, just before the first state of emergency was declared. March, June, September, and December represent spring, summer (hot season), autumn, and winter (cold season), respectively [19]. Additionally, seasonal glycemic variations were analyzed by comparing the median HbA1c levels determined at each visit and differences across the study period. Moreover, we conducted stratified analyses of seasonal variations in glycemic control by categorizing the patients into two age groups: older (≥ 70 years) and younger (< 70 years), and two lifestyle groups: with or without lifestyle changes (food intake or physical activity).

### Statistical analysis

Quantitative data are presented as mean ± standard deviation for normally distributed variables and as median plus interquartile range (IQR) and range for non-normally distributed variables. Normally distributed variables compared with variables showing a non-normal distribution are presented as median plus IQR and range. The normal distribution of each variable was confirmed using the Shapiro–Wilk test (Supplementary Table S1). Comparisons of categorical variables, such as sex, complications, and drugs in each group, were performed using Fisher’s exact test. Continuous variables were analyzed using the Wilcoxon signed-rank test and Kruskal–Wallis test for 2 and ≥ 3 paired samples, respectively, and analysis of variance (ANOVA). Statistical significance was set at *P* < 0.05. All analyses were performed using JMP Pro 14 software (SAS Institute Inc., Cary, NC, USA).

Power analysis was performed to confirm whether the number of participants was sufficient to collect the baseline data. The mean baseline and standard deviation of HbA1c (NGSP) were 7.86% and 1.08%, respectively (Table 2). Based on previous studies on the clinical significance [20] and effects of lifestyle modifications [7], an improvement of 0.5% in HbA1c level was considered clinically significant. The required number of participants when the α and β error values were set as 0.05 and 0.2, respectively, was 65. Thus, 86 participants were considered sufficient for this study.

**Table 2:** Seasonal variations in clinical parameters in 2019, 2020, and 2021 (*n* = 86)

Parameters	2019			2020			2021			Comparison of the data of the 3 years, *P*-value	
	March	September, Change value^†^	*P*-value	March	September, Change value^†^	*P*-value	March	September, Change value^†^	*P*-value	March^‡^	Change value^‡^
HbA1c (%, NGSP) [mmol/mol, IFCC]	7.7 (7.2–8.3) [60.6 (55.2–67.2)]	− 0.4 (− 0.7 to 0.0) [− 4.4 (− 7.7 to 0.0)]	** < 0.001**	7.4 (6.9–7.9) [56.8 (51.9–62.8)]	− 0.2 (− 0.6 to 0.1) [− 2.2 (− 6.6 to 1.1)]	** < 0.001**	7.5 (7.1–8.0) [58.5 (54.1–63.9)]	− 0.3 (− 0.7 to 0.0) [− 3.3 (− 7.7 to 0.0)]	** < 0.001**	0.065	0.77
Body weight (kg)	64.2 (55.8–71.5)	− 0.3 (− 1.5 to 0.5)	**0.013**	65.3 (55.1–72.6)	− 0.7 (− 1.8 to 0.4)	** < 0.001**	63.8 (55.3–71.9)	− 0.5 (− 1.9 to 0.5)	**0.009**	0.91	0.61
BMI (kg/m^2^)	24.5 (22.1–26.9)	− 0.1 (− 0.7 to 0.2)	**0.016**	24.7 (22.1–26.9)	− 0.3 (− 0.7 to 0.2)	** < 0.001**	24.2 (22.2–26.9)	− 0.2 (− 0.7 to 0.2)	**0.006**	0.88	0.68
AST (U/L)	21.0 (17.0–26.0)	0.0 (− 3.0 to 2.0)	0.79	20.0 (17.0–24.5)	0.0 (− 2.0 to 3.0)	0.44	21.0 (18.0–25.0)	− 1.0 (− 3.8 to 3.0)	0.33	0.94	0.41
ALT (U/L)	17.5 (13.0–22.3)	1.0 (− 3.0 to 5.0)	0.21	18.0 (13.0–25.0)	1.0 (− 1.0 to 4.0)	0.13	19.0 (14.0–26.0)	0.0 (− 3.0 to 3.0)	0.40	0.95	0.17
γ-GT (U/L)	25.5 (16.8–40.3)	1.0 (− 3.0 to 3.0)	0.29	24.5 (17.0–40.0)	0.0 (− 3.0 to 2.0)	0.78	23.0 (15.0–39.0)	0.0 (− 3.0 to 3.0)	0.42	0.71	0.88
BUN (mg/dL)	16.7 (13.8–20.7)	0.25 (− 2.3 to 3.0)	0.41	17.3 (13.7–20.8)	− 0.7 (− 3.1 to 2.1)	0.78	17.4 (14.3–20.8)	0.0 (− 1.6 to 2.8)	0.25	0.98	0.79
Creatinine (mg/dL)	0.78 (0.67–1.02)	0.0 (0.0–0.1)	**0.026**	0.8 (0.7–1.0)	0.0 (0.0–0.1)	**0.005**	0.8 (0.7–1.0)	0.0 (0.0–0.1)	0.054	0.71	0.77
eGFR (mL/min/1.73 m^2^)	67.0 (53.3–81.4)	− 0.9 (− 6.5 to 3.1)	0.068	68.9 (54.2–81.0)	− 3.5 (− 7.8 to 1.2)	** < 0.001**	66.9 (51.7–78.5)	− 2.0 (− 6.9 to 1.2)	**0.031**	0.67	0.62
Triglyceride (mg/dL)	139 (88.3–207.5)	2.0 (− 46.8 to 41.3)	0.95	145 (103–183)	0.0 (− 19.3 to 32.0)	0.47	133 (88–195)	− 2.0 (− 29.3 to 21.0)	0.83	0.97	0.90
High-density lipoprotein (mg/dL)	55.9 (46.6–63.6)	− 2.0 (− 6.0 to 0.5)	** < 0.001**	55.2 (46.4–65.3)	− 0.8 (− 5.9 to 3.1)	0.25	52.6 (45.9–61.2)	− 1.3 (− 3.9 to 3.1)	0.18	0.52	0.15
Low-density lipoprotein (mg/dL)	114.9 (96.8–134.8)	− 5.2 (− 16.9 to 2.5)	**0.006**	110.1 (83.0–129.9)	− 0.7 (− 15.4 to 7.7)	0.24	104.2 (82.0–123.9)	− 1.5 (− 13.4 to 9.5)	0.37	**0.027**	0.065

HbA1c levels are presented as NGSP values (%), with IFCC values (mmol/mol) in brackets. Values are expressed as median (interquartile range). ^†^, Change values compared with the data obtained in last March are presented. ^‡^, Kruskal–Wallis test was used to analyze parameters obtained in the 3-year period (2019–2021). HbA1c level and BMI showed significant difference between March and September every year but no significant difference in variations during the study period. *P*-values of < 0.05 indicated significance (boldface)

Abbreviations: *AST* aspartate aminotransferase, *ALT* alanine transaminase, *BUN* blood urea nitrogen, *eGFR* estimated glomerular filtration rate, *γ-GT* γ-glutamyl transferase, *HbA1c* hemoglobin A1c, *NGSP* National Glycohemoglobin Standardization Program

### Patient and public involvement statement

The design, conduct, reporting, and dissemination plans of the present study were not influenced by the patients or the public.

## Results

### Baseline characteristics of the participants

The baseline characteristics of the 86 participants are listed in Table 1. All participants were Japanese. None of the participants had developed COVID-19. The mean age of the participants was 69.6 ± 9.2 years, and there were 57 men (66.3%). The median duration of DM was 13.5 (IQR:9–23; range:1–50) years. Current smokers and alcohol drinker (≥ 4 days/week) were 31 (36.0%) and 22 (25.6%), respectively, and none of them had a low income (< 150,000 JPY/M). Regarding complications associated with DM, neuropathy, retinopathy, and nephropathy were observed in 43 (50.0%), 22 (25.6%), and 33 (38.4%) patients, respectively. Hypertension, dyslipidemia, and cardiovascular disease were observed in 57 (66.3%), 49 (57.0%), and 24 (27.9%) patients, respectively. Among the 25 patients (29.1%) who stated that their overall lifestyle had changed during the COVID-19 pandemic, 12 (14.0%) increased their dietary intake and 20 (23.3%) decreased their physical activity level (Table 1). The universal health insurance system supports patients in accessing hospitals in Japan and covers all patients in this study.

### Seasonal variations in glycemic control and other clinical parameters before and after the spread of COVID-19

**Fig. 2:**
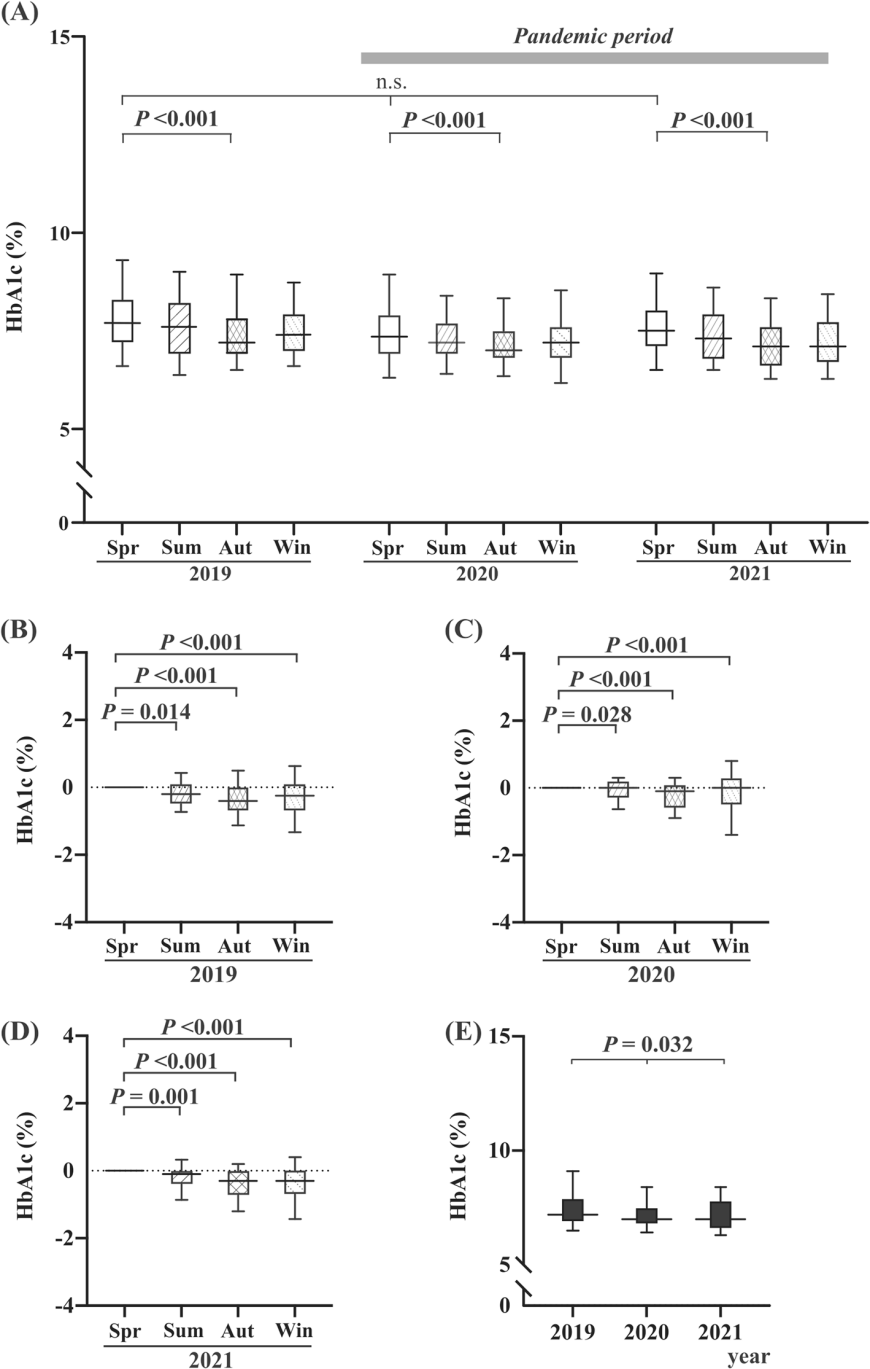
Comparison of HbA1c levels throughout the study period (March 2019 and December 2021). (**A**) Changes in HbA1c levels during the study period. The difference between spring (Spr: March), summer (Sum: June), autumn (Aut: September), and winter (Win: December) in HbA1c levels are presented in (**B**) 2019, (**C**) 2020, and (**D**) 2021. Each value is presented in comparison with the levels in the spring (March) of each year. In 2020, median HbA1c levels significantly decreased from 7.35% [56.8 mmol/mol] in spring (March) to 7.00% [53.0 mmol/mol] in autumn (September) (*P* < 0.001) during the peak of COVID-19 prevalence in Japan. In 2019, the median HbA1c level significantly decreased from 7.70% [60.6 mmol/mol] in spring (March) to 7.20% [55.2 mmol/mol] in autumn (September) (*P* < 0.001). No significant difference in HbA1c levels was observed between 2020 and 2019. Additionally, HbA1c levels in autumn (September) for each year are presented. HbA1c levels in the spring (March) of 2019, 2020, and 2021 did not show any significant differences (*P* = 0.083). The horizontal lines and open squares represent the median and interquartile range, respectively, at each point. Vertical bars at each point indicate the range. The Wilcoxon signed-rank test was used to compare spring (March) and autumn (September) data for each year, and analysis of variance was used to compare spring (March) data. Statistical significance was set at *P* < 0.05. Abbreviations: Aut, autumn; n.s., not significant; Spr, spring; Sum, summer; Win, winter

The mean HbA1c levels, body weight, and BMI continuously exhibited a significant difference (low in September and high in March) during the 3-year period. However, low-density lipoprotein levels showed no seasonal variations in 2020 and 2021 but showed some changes in 2019 (Table 2 and Fig. 2A). No other parameters showed continuous seasonal variation between spring (March) and autumn (September) during the study period (Table 2).

**Fig. 3:**
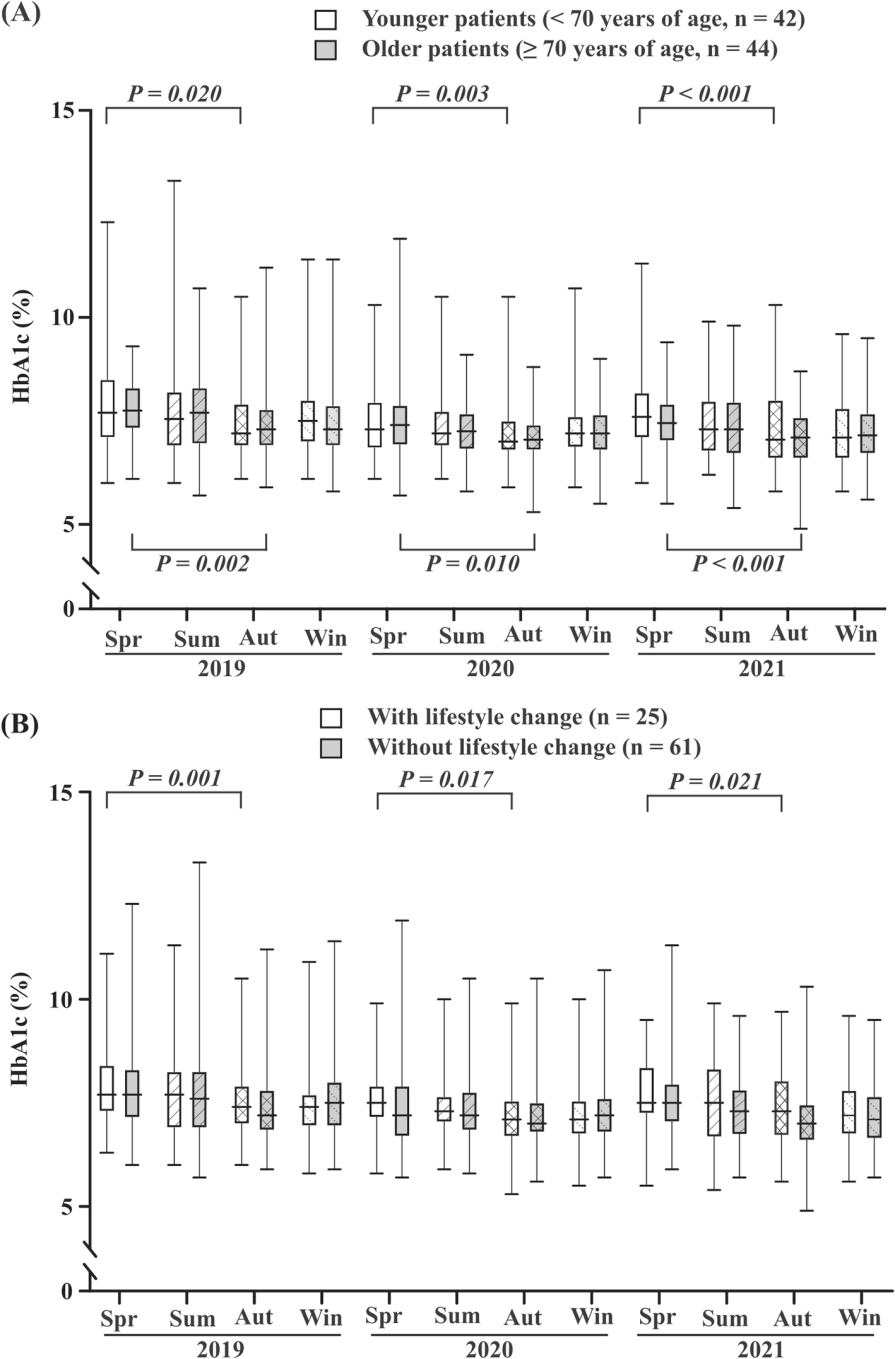
Seasonal glycemic variation of HbA1c levels; stratified analysis for age or lifestyle changes. Seasonal glycemic variation in HbA1c levels (**A**) in older (age: ≥ 70 years; *n* = 44) or younger (age: < 70 years; *n* = 42) patients and (**B**) with (*n* = 25) or without (*n* = 61) lifestyle changes in spring (Spr: March), summer (Sum: June), autumn (Aut: September), and winter (Win: December) during the 3-year study period (2019–2021). The horizontal lines in the squares represent the medians at each point. Squares and vertical bars indicate the interquartile range and range, respectively, at each point. Both older and younger patients with type 2 diabetes mellitus demonstrated similar seasonal changes in HbA1c levels. Additionally, this variation was preserved during the COVID-19 outbreak

The median HbA1c levels in spring (March) and autumn (September) were 7.86% [61.2 mmol/mol] and 7.48% [57.4 mmol/mol] in 2019 (*P* < 0.001), 7.50% [57.7 mmol/mol] and 7.17% [54.2 mmol/mol] in 2020 (*P* < 0.001) and 7.61% [58.3 mmol/mol] and 7.19% [53.8 mmol/mol] in 2021 (*P* < 0.001), indicating that the seasonal variation in HbA1c levels was preserved. In this context, the variation in 2020 and 2021 (during the COVID-19 pandemic) did not vary significantly from that in 2019 (Table 2 and Fig. 2A–D). Additionally, HbA1c levels in spring (March) did not show a significant difference among the 3 years (2019, 2020, and 2021; Table 2 and Fig. 2E). Furthermore, no significant differences were observed in the HbA1c values in summer (June) and winter (December) for 3 years, although HbA1c levels in autumn (September) showed significant differences among 3 years (Fig. 2E). Age-stratified analysis of the participant data revealed similar seasonal variations between older and younger patients (Fig. 3A). Seasonal variation was revealed in both patients with and without diet and exercise lifestyle changes (Fig. 3B).

### Changes in medical treatments during the study period

Metformin was the most frequently administered class of drugs (33 patients, 39.8%), followed by dipeptidyl peptidase-IV inhibitor (31 patients, 36.1%). Insulin injection was administered to 22 patients (25.6%), and the median total daily insulin dose was 24.5 (IQR:19.3–37.5; range 10–103) units (Supplementary Table S2). The use of metformin, dipeptidyl peptidase-IV inhibitors, sodium glucose cotransporter 2 inhibitors, and glucagon-like peptide-1 receptor agonists increased during the study period (Supplementary Table S2). Comparing the data from March and September of each year, the use of dipeptidyl peptidase-IV inhibitors increased in 2019, but there was no difference in the use of other oral hypoglycemic agents.

## Discussion

COVID-19 had no effect on seasonal variations in the glycemic profiles of patients with type 2 DM in this study. Two reasons may explain this finding. First, there were few restrictions on outdoor activities as COVID-19 was not severe in this region. Second, the lifestyle of older patients after the spread of COVID-19 did not change because their lifestyle before the pandemic was already consistent with their avoidance of 3Cs. In other words, the outcomes of the present study showed that glycemic control in older patients with DM was not affected by physical distancing, although it could be affected in the long-term. Therefore, lifestyle modifications due to COVID-19 may be age-dependent because children and adolescents are forced to modify their own lifestyles [21, 22]. However, in this study, there were no differences between the older (≥ 70 years) and younger (< 70 years) groups.

After the spread of COVID-19, HbA1c levels changed to the same degree as they did in the previous year—before the pandemic. In contrast, COVID-19 has been reported to adversely affect lifestyle elements, including diet and exercise, in the general population [23]. Disturbance in eating habits, decline in physical activity level, and decrease in the frequency of blood glucose self-monitoring have been reported in patients with type 2 DM [21]. Hence, it has been suggested that COVID-19 worsens glycemic control in patients with DM. The effects of the COVID-19 pandemic on T2DM management remain controversial. Recent studies have reported COVID-19 pandemic-associated worsening of glycemic management [24, 25]. Japanese receipt data reported a 5.86% decrease in the number of hospital visits of patients with DM after the COVID-19 pandemic compared to that before the pandemic [26]. Conversely, HbA1c levels among low-income individuals decreased in the United States during the COVID-19 pandemic when access to healthcare was reduced [27].

In this study, BMI was found to have seasonal variations similar to HbA1c; however, the pandemic had no effect on such variations. This finding was similar to the fact that lockdowns in Spain due to COVID-19 led to no overall anthropometric changes [28]. Body weight reduction related to reduction in body fat accumulation leads to improved glycemic profiles through a reduction in insulin resistance in patients with type 2 DM [29]. However, skeletal muscle atrophy should also be noted as a cause of body weight reduction, which is important because older patients comprised a large percentage of the participants in this study. Following the Great East Japan Earthquake, muscle weakness was reported to be a problem in older people who were forced to live in evacuation zones [30]. Short-term disuse has been suggested as a risk factor for skeletal muscle atrophy in older patients [31]. Based on the results of previous studies [32], clinicians should be aware that sarcopenia is a risk factor for body weight reduction. Furthermore, seasonal variations overcome lifestyle-related reductions in physical activity, which may result in loss of body muscle volume.

Seasonal variations in HbA1c levels in patients with type 2 DM may not be eliminated by COVID-19-associated lifestyle modifications. Circadian rhythms, which are synchronized with light and temperature [3, 33], are also closely associated with glucose metabolism, and their disturbances interrupt glucose metabolism [34]. Changes in both temperature and seasonal lifestyle are considered key factors contributing to seasonal glycemic variation. People living in areas with lower temperature changes show lower seasonal fluctuations in HbA1c levels [35, 36]. The present study revealed that seasonal variations in HbA1c levels were not affected by lifestyle modifications due to COVID-19. Additionally, COVID-19 infection exacerbated DM profiles based on the prohibition of regular treatments for DM and dexamethasone administration [37]; however, none of the participants had been infected with symptomatic COVID-19 during the study period. Information on seasonal variations in glycemic control independent of lifestyle modifications may improve the management of patients with type 2 DM.

This study had three limitations. First, the small number of participants in the current study warrants further large-scale studies to validate the findings. Second, this was a single-center study, and we could not conclude whether our results could be generalized to other countries. Third, we could not exclude the influence of medications in this retrospective study. Future international prospective studies may clarify the physiology of seasonal glycemic variation.

## Conclusions

The COVID-19 pandemic in this area did not prevent seasonal variations in the glycemic and BMI profiles of older patients with type 2 DM, who were permitted to receive regular treatments and did not develop COVID-19 during the study period. In Japan, seasonal variations substantially affect the glycemic profile and BMI of patients with type 2 DM. Thus, these variations in glycemic profile and BMI in patients with type 2 DM should be regulated beyond lifestyle modifications due to COVID-19 in Japan. Even if the pandemic alters the level of physical activity in patients with type 2 DM, more studies on the effects of seasonal variations on human physical functions may help develop strategies for better management of patients with type 2 DM.

### Supplementary Information

Supplementary Material 1.

## Data Availability

The datasets generated and/or analyzed during the current study are not publicly available due to some restrictions that applied by the ethical committee but are available from the corresponding author on reasonable request.

## Abbreviations

ANOVA: Analysis of variance
BMI: Body mass index
COVID-19: Coronavirus disease 2019
DM: Diabetes mellitus
HbA1c: Hemoglobin A1c
IQR: Interquartile range
NGSP: National Glycohemoglobin Standardization Program
