# Supplementary material for: Preserved seasonal variation in glycemic control in patients with type 2 diabetes mellitus during COVID-19: a 3-year-long retrospective cohort study in older adults in Japan
Source: BMC Endocr Disord. 2024 May 17;24:70. doi: 10.1186/s12902-024-01602-8 (PMC11100128; doi:10.1186/s12902-024-01602-8)
Supplement: Supplementary file 1 — Supplementary Material 1. [file 12902_2024_1602_MOESM1_ESM.docx]

| **Supplementary Table S1. The results of Shapiro–Wilk test for the parameters evaluated in this study** | | | | | | | | | | | |
| --- | --- | --- | --- | --- | --- | --- | --- | --- | --- | --- | --- |
| **Parameters** | **2019** | | |  | **2020** | | |  | **2021** | | |
|  | **March** | **September** | **change value** |  | **March** | **September** | **change value** |  | **March** | **September** | **change value** |
| **Age Total** | 0.15 |  |  |  |  |  |  |  |  |  |  |
| **Men** | 0.31 |  |  |  |  |  |  |  |  |  |  |
| **Women** | 0.098 |  |  |  |  |  |  |  |  |  |  |
| **Duration of DM** | 0.001 |  |  |  |  |  |  |  |  |  |  |
| **Body weight** | 0.42 | 0.004 | <0.001 |  | 0.002 | 0.006 | <0.001 |  | 0.082 | 0.090 | <0.001 |
| **BMI** | 0.012 | <0.001 | <0.001 |  | <0.001 | <0.001 | <0.001 |  | <0.001 | <0.001 | 0.010 |
| **HbA1c level** | 0.026 | <0.001 | <0.001 |  | <0.001 | <0.001 | <0.001 |  | 0.004 | 0.008 | 0.039 |
| **AST level** | <0.001 | <0.001 | 0.002 |  | <0.001 | <0.001 | <0.001 |  | <0.001 | <0.001 | <0.001 |
| **ALT level** | <0.001 | <0.001 | 0.010 |  | <0.001 | <0.001 | <0.001 |  | <0.001 | <0.001 | <0.001 |
| **γ-GT level** | <0.001 | <0.001 | <0.001 |  | <0.001 | <0.001 | <0.001 |  | <0.001 | <0.001 | <0.001 |
| **BUN level** | <0.001 | <0.001 | 0.86 |  | <0.001 | <0.001 | 0.005 |  | <0.001 | <0.001 | <0.001 |
| **Creatinine level** | <0.001 | <0.001 | <0.001 |  | <0.001 | <0.001 | <0.0001 |  | <0.001 | <0.001 | <0.001 |
| **eGFR** | 0.042 | 0.029 | 0.13 |  | 0.061 | 0.016 | 0.32 |  | 0.12 | 0.12 | <0.001 |
| **Triglyceride level** | <0.001 | <0.001 | <0.001 |  | <0.001 | 0.021 | <0.001 |  | <0.001 | <0.001 | <0.001 |
| **HDL-cholesterol level** | 0.007 | 0.39 | 0.55 |  | 0.074 | 0.22 | 0.063 |  | <0.001 | <0.001 | 0.56 |
| **LDL-cholesterol level** | <0.001 | 0.93 | <0.001 |  | 0.14 | 0.082 | 0.99 |  | 0.78 | 0.099 | 0.61 |
| **Insulin dose** |  |  |  |  |  |  |  |  |  |  |  |
| Total | <0.001 | <0.001 |  |  | 0.001 | 0.001 |  |  | <0.001 | <0.001 |  |
| Basal | <0.001 | <0.001 |  |  | 0.18 | 0.068 |  |  | 0.11 | 0.15 |  |
| Bolus | 0.059 | 0.044 |  |  | 0.014 | 0.025 |  |  | 0.035 | 0.002 |  |

*P* values of the Shapiro–Wilk test are presented. *P* <0.05 was considered significant and show non-normal distribution.

| **Supplementary Table S2. Diabetes medications in each phase (n = 86)** | | | | | | | | | | | |
| --- | --- | --- | --- | --- | --- | --- | --- | --- | --- | --- | --- |
| **Drugs** | **2019** | |  | **2020** | |  | **2021** | |  | *P*-value^†^ |  |
|  | March | September |  | March | September |  | March | September |  |  |  |
| **Insulin dose**, n (%) | 22 (25.6) | 22 (25.6) |  | 25 (29.1) | 23 (26.7) |  | 25 (29.1) | 24 (27.9) |  | 0.64 |  |
| Total dose, units per day | 24.5 (18.8–39.3) | 23.5 (18.8–37.8) |  | 21.0 (17.0–35.5) | 21.0 (15.5–37.5) |  | 21.0 (15.5–35.5) | 19.0 (12.5–33.5) |  | 0.62 |  |
| Basal, units per day | 14.4 (10.0–22.3) | 14.4 (10.0–22.0) |  | 14.7 (10.0–21.0) | 14.7 (9.5–22.5,) |  | 14.7 (9.5–20.1) | 14.0 (9.0–20.0) |  | 0.55 |  |
| Bolus, units per day | 15.9 (6.1–30.0) | 15.9 (6.1–30.0) |  | 15.8 (6.9–28.0) | 15.8 (7.7–28.5) |  | 15.0 (8.2–31.5) | 8.5 (2.0–25.0) |  | 0.99 |  |
| **GLP-1 receptor agonist**, n (%) | 3 (3.5) | 3 (3.5) |  | 5 (5.8) | 4 (4.7) |  | 6 (7.0) | 8 (9.3) |  | **0.068** |  |
| **Metformin**, n (%) | 35 (40.1) | 46 (53.5) |  | 52 (60.5) | 52 (60.5) |  | 55 (64.0) | 56 (65.1) |  | **<0.001** |  |
| **Sulfonylurea**, n (%) | 32 (37.2) | 30 (34.9) |  | 25 (29.1) | 26 (30.2) |  | 26 (30.2) | 25 (29.1) |  | 0.21 |  |
| **Glinide**, n (%) | 9 (10.5) | 10 (11.6) |  | 10 (11.6) | 9 (10.5) |  | 9 (10.5) | 9 (10.5) |  | 0.87 |  |
| **DPP-IV inhibitor**, n (%) | 31 (36.1) ^‡^ | 44 (51.2) ^‡^ |  | 48 (55.8) | 57 (66.3) |  | 55 (64.0) | 57 (66.3) |  | **<0.001** |  |
| **SGLT-2 inhibitor**, n (%) | 22 (25.6) | 31 (36.1) |  | 35 (40.7) | 36 (41.9) |  | 36 (41.9) | 39 (45.4) |  | **0.007** |  |
| **Pioglitazone**, n (%) | 3 (3.5) | 3 (3.5) |  | 3 (3.5) | 3 (3.5) |  | 3 (3.5) | 3 (3.5) |  | 1.00 |  |
| **α-glucosidase inhibitor**, n (%) | 11 (12.8) | 14 (16.3) |  | 12 (14.0) | 12 (14.0) |  | 11 (12.8) | 9 (10.5) |  | 0.49 |  |
| Insulin doses are presented as median (interquartile range). †, Total, basal, and bolus insulin doses were analyzed using the Kruskal–Wallis test. Other parameters were analyzed using the Cochran Armitage trend test. *P-*values of <0.05 were considered significant (boldface). ‡, the use of DPP-IV inhibitors increased in September 2019 compared with that in March 2019 (*P* = 0.044). Abbreviations: GLP-1, glucagon-like peptide-1; DPP-IV, dipeptidyl peptidase-IV; SGLT-2, sodium glucose cotransporter. | | | | | | | | | | | |
